# The Use of Fear versus Hope in Health Advertisements: The Moderating Role of Individual Characteristics on Subsequent Health Decisions in Chile

**DOI:** 10.3390/ijerph17239148

**Published:** 2020-12-07

**Authors:** Pablo Farías

**Affiliations:** Departamento de Administración, Facultad de Economía y Negocios, Universidad de Chile, Santiago 8330015, Chile; pfarias@fen.uchile.cl

**Keywords:** healthy eating intention, physical activity intention, emotions, aversive state, reinforcement, social support, self-concept

## Abstract

No studies have addressed the way the effectiveness of fear and hope advertisements differs across differently characterized individuals. The present study aims to find out in which situations related to different individual characteristics do fear and hope advertisements work as tools in generating healthy eating intention and physical activity intention. This study conducted an experiment using 283 adults from Chile. The results suggest that fear versus hope appeals in health advertisements have a more positive influence on healthy eating intention. The results suggest that the effect of fear advertisements on healthy eating intention is positively moderated by the frequency of fast food consumption and is negatively moderated by self-efficacy. The results suggest that fear versus hope appeals in health advertisements have no main effect on physical activity intention. However, the results suggest that the effect of fear advertisements on physical activity intention is positively moderated by perceived body weight and past healthy eating behavior and is negatively moderated by subjective norms. The results indicate that when making health advertising, homogenous messages are not persuasive for heterogeneous audiences. The present study results suggest that fear and hope advertisements should be delivered considering the individual characteristics identified in the present study.

## 1. Introduction

Physical activity and healthy eating are part of the solution for the ongoing obesity epidemic [1,2]. In the last decade, national policy initiatives focused on physical activity and healthy eating have spread in Latin American countries. Programs aimed at promoting healthy lifestyles by addressing eating behaviors and physical activity are at the center of every government’s agenda. Despite these efforts, in Latin America, the prevalence of overweight and obese adults has increased markedly in the last three decades [3,4,5,6,7].

Emotions play a significant role in advertising [8,9]. The emotions of fear and hope could stimulate individuals to make health-related decisions [10,11,12]. In the U.S., Krishen and Bui [13] reported that fear-based framing of health messages could lead to positive decision intentions and, consequently, help people make better health-related decisions. Fear is a powerful emotion in getting individuals to change their behavior. Advertising, especially health advertising, is meant to be effective and aims to change individuals’ behaviors towards their health. Thus, fear advertising may be considered a useful tool for changing people’s attitudes towards healthy eating and participating in physical activity [10,14]. The frequency of fear appeals in advertisements varies across countries and cultures [15,16]. Unfortunately, in Latin America, not enough is known about the potential that fear-mongering could have in health advertisements. There is no research analyzing its effectiveness in the region, and fear-mongering advertising has seldom been employed. Consequently, this research study’s first objective is to examine the effects of fear-based framing of health advertisements on healthy eating intention and physical activity intention among adults in Chile.

A major limitation of the Krishen and Bui [13] study was that they treated subjects as a homogenous group. The way recipients process messages from fear appeals could be affected by their particular characteristics [17,18]. No studies have addressed the way the effectiveness of fear-based advertisements differs across differently characterized individuals, or the circumstances under which fear-based advertisements could potentially influence health-related choices. In fact, Krishen and Bui [13] recommend that future research includes the moderating roles of self-esteem, self-control, and other individual characteristics on subsequent health decisions. For these reasons, this study’s second objective is to incorporate the individual characteristics (frequency of fast food consumption, body weight, past behavior, subjective norms, social influences regarding physical activity, self-efficacy, self-esteem, and self-control) as moderators. This research aims to identify the situations related to different individual attributes in which fear-mongering advertisements work as a useful tool to elicit behavior changes. Table 1 summarizes the identified hypotheses to achieve both research objectives.

### 1.1. Fear Versus Hope Appeals in Health Advertisements

The frequency of fear appeals in advertisements varies across countries and cultures [15,16]. Fear appeals are more frequently used in Canada and China than in France [15]. This resonates with the cultural value of uncertainty avoidance, a characteristic more present in France than in Canada or China [15,19]. Previous studies have found evidence that fear works [20]. Krishen and Bui [13] reveal that, in the U.S., fear-based framing of health messages can direct individuals to positive decision intentions, thus helping them make better-planned health-related decisions. The question then arises: do cultural aspects influence the effects of fear versus hope appeals in health advertisements? Latin America has higher uncertainty avoidance levels than the U.S. [19,21]. The uncertainty avoidance score expresses the extent to which the members of a culture feel threatened by ambiguous or unknown situations and have created beliefs and institutions to try avoiding these circumstances [19]. Given that cultures with high uncertainty avoidance levels have beliefs and institutions to deal with fear, Matsumoto [22] suggests that people may tend not to recognize fear or may attenuate attributions of its intensity when sensed or expressed. As there is no information on the potential of fear appeals in health advertisements in Latin America, to examine the fear-priming effects, the first hypothesis is proposed:

**Hypothesis** **1.** When subjects view a fear advertisement in Chile, they will be more likely to report (a) greater healthy eating intention and (b) greater physical activity intention, than when they view a hope advertisement.

### 1.2. The Moderating Role of Individual Characteristics

A major limitation of Krishen and Bui’s [13] study was that they treated subjects as a homogenous group. The way recipients process messages from fear appeals could be affected by their particular characteristics [17]. No research has studied the circumstances under which fear advertisements can potentially influence health-related decisions and how their effectiveness varies across individuals. This study aims at figuring out if different reactions from people with different characteristics will affect the results of fear advertising. Krishen and Bui [13] suggest that traits such as self-esteem and self-control, among others, should be considered in future research.

#### 1.2.1. Fear Appeals and Avoiding an Aversive State

Fast food is generally low cost and the promotion is active, referring to consumers’ social surroundings and behaviors [23,24]. Frequent fast food consumption has been linked to obesity, diabetes, hypertension, and heart disease [25,26]. In a hope environment, a favorable outcome could occur (e.g., healthy living), whereas, in a fear environment, an unfavorable outcome could be avoided or even resolved (e.g., death) [27]. The emotion of fear could stimulate individuals to deal with the causes of that emotion (fast food consumption) [10]. Fear engenders a desire to escape from or avoid an aversive state [28]. As a primary aversive emotion, fear arises in situations of menace to the organism (i.e., the individual) and enables them to respond to them adaptively [29]. Through fear, individuals with frequent fast food consumption may have a greater intention to eat healthily and do physical activity in order to escape from this aversive state [28,29], that is, the negative effects (e.g., obesity, diabetes, hypertension, heart disease) associated with frequent consumption of fast food. [25,26]. Consequently, the effects of fear (vs. hope) appeals in health advertisements on healthy eating intention and physical activity intention could be stronger among individuals having higher (e.g., once a day, more than once a day) rather than lower (e.g., occasionally, never) frequency of fast food consumption. Hence:

**Hypothesis** **2.** The effects of fear (vs. hope) appeals in health advertisements on (a) healthy eating intention and (b) physical activity intention are stronger among individuals who consume fast food more frequently than others.

Researchers have shown that overweight individuals are more likely to be present-biased [30]. Present-biased individuals tend to value more immediate rewards than rewards that would happen over time [31]. In the case of overweight individuals, the immediate action of eating healthy or doing physical activity would be relatively more appealing if they feel rewarded by doing so. The reward could be psychological or material; it is highly valued as long as it is immediate. Fear-based health advertising could thus have a stronger effect on overweight individuals as the promoted effects would be immediate (e.g., avoid hypertension and heart diseases). Hence:

**Hypothesis** **3.** The effects of fear (vs. hope) appeals in health advertisements on (a) healthy eating intention and (b) physical activity intention are stronger among individuals who are overweight.

#### 1.2.2. Fear Appeals and Reinforcement

Fear appeals elicit increased brain processing and more robust recall [32]. The effects of fear in terms of past behavior have been researched, especially in individual decision-making leading to some sort of pain. If an individual remembers a painful time in their life, they are likely to act against similar events in the future [33]. Fear appeals could be used in a way that allows fear of reinforcing appropriate past behavior [34]. Fear could thus work as reinforcement in making healthy decisions. Hence:

**Hypothesis** **4.** The effects of fear (vs. hope) appeals in health advertisements on (a) healthy eating intention and (b) physical activity intention are stronger among individuals who have higher past healthy behaviors.

#### 1.2.3. Hope Appeals and Social Support

Social support is the individual’s support behaviors from people in their social network, which enhance their mood, performance, and activities. Social support is a significant factor affecting a person’s eating and physical activity intentions and behaviors [35,36,37,38]. The effects of subjective norms and social influences regarding healthy eating intentions and physical activity intentions could be enhanced if the individual is motivated more by hope (positive goals are expected) rather than fear (negative outcomes should be avoided). Therefore, hope (vs. fear) appeals could be more effective in cases where individuals have high subjective norms and social influences regarding healthy eating intentions and physical activity intentions. Hence:

**Hypothesis** **5.** The effects of hope (vs. fear) appeals in health advertisements on (a) healthy eating intention and (b) physical activity intention are stronger among individuals who have higher subjective norms.

**Hypothesis** **6.** The effects of hope (vs. fear) appeals in health advertisements on (a) healthy eating intention and (b) physical activity intention are stronger among individuals who have more social influences regarding physical activity.

#### 1.2.4. Hope Appeals and Self-Concept

Hope as an emotion emerges when a concrete positive goal is envisioned. It comprises a cognitive element of expecting and an affective feel-good element about the expected events or outcomes [39,40]. Hope is a positive motivational state. Hopeful individuals are more intrinsically motivated and enjoy pursuing goals [41]. In order to reach such goals and feel hope, an individual must be rather determined and goal-oriented and possess some self-efficacy, self-esteem, and self-control.

As concluded by Snyder [42], hope messaging can contribute to goal-oriented behaviors geared towards improving states already in satisfactory levels rather than in other unsatisfactory ones. Hope requires mental representations of positively valued abstract future situations, and, more specifically, it requires setting goals, planning how to achieve them, using imagination, creativity, cognitive flexibility, and mental exploration of novel situations [29]. An individual’s beliefs about their capabilities to perform well in situations that affect their lives is denoted by self-efficacy. It rules how individuals feel, think, motivate themselves, and behave in such cases. People with high self-efficacy are determined and motivated, whereas people with low self-efficacy doubt their capabilities, are not committed to pursuing their tasks and goals, and see difficulties as obstacles and challenging tasks as personal threats [43,44]. For example, Mowen et al. [45] comment that individuals with higher self-efficacy experience lower fear levels.

For intrinsically motivated people (e.g., high in self-esteem or self-control), hope appeals in health advertisements may work as a better motivator than fear appeals. People who are more likely to be externally motivated (e.g., individuals low in self-esteem or self-control) may find fear more appealing than hope, due to the stronger emotional effects fear has on an individual. Hence:

**Hypothesis** **7.** The effects of hope (vs. fear) appeals in health advertisements on (a) healthy eating intention and (b) physical activity intention are stronger among individuals who have higher levels of self-efficacy.

**Hypothesis** **8.** The effects of hope (vs. fear) appeals in health advertisements on (a) healthy eating intention and (b) physical activity intention are stronger among individuals who have higher levels of self-esteem.

**Hypothesis** **9.** The effects of hope (vs. fear) appeals in health advertisements on (a) healthy eating intention and (b) physical activity intention are stronger among individuals who have high levels of self-control.

## 2. Materials and Methods

### 2.1. Procedures

Data were collected using an online self-administered questionnaire sent to a convenience sample of adults from Chile. Some measures included in the questionnaire may represent a personally sensitive issue. Respondents may not have wanted to face an interviewer and would like to have completed the survey alone. In order to obtain sensitive information (e.g., perceived body weight, self-esteem), to offer perceived respondent anonymity (i.e., respondents’ perceptions that the interviewer or researcher will not discern their identities), and to avoid social desirability (i.e., for respondents to answer what they feel to be acceptable in front of others), an invitation to participate in an anonymous online survey on health advertisements was emailed to a database of people over 18 years of age living in Chile. The final sample size was 283 adults (response rate = 23.2%). All response data were anonymous. Participants were randomly assigned one of two events (fear or hope advertisement). Subjects were told to watch a video (fear or hope advertisement) for about two minutes. Then, they had to complete an online questionnaire.

### 2.2. Stimuli

Fear and hope advertisements had the same modality (audiovisual) and a similar length (102 and 113 s, respectively). The fear advertisement is an edited version of a video released by the Children’s Healthcare of Atlanta. Through Jim’s life, the video starts with him lying on a hospital bed after suffering a heart attack at the age of 32, then rewinds to expose the patterns of an unhealthy and sedentary lifestyle that lead him to that point. In different stages of his life, Jim is shown eating unhealthy foods, sitting around watching television, and playing computer games. In the edited version, sentences were modified to communicate that adults should change their habits to improve their own health.

The hope advertisement is an edited version of a video released by Coke. The video shows that they published advertisements in a newspaper to promote weight loss pills (magic pills). Without mentioning the brand, the advertisement said the first individuals to call would win a free sample of magic pills. From all the calls, three individuals were chosen to receive the magic pills. Nonetheless, they were not expecting the many obstacles on their way there such as staircases, barking dogs, damsels in distress, and grannies with packages to carry. In the end, the participants find out that it was all a well-staged, provocative, and inspiring prank to encourage them to lose weight. They were shown the tapings of themselves to learn that every bit of effort and activity in their routine contributes more to their weight and fitness than a magical pill. The video was shortened by removing some obstacles. The mention of Coke was also removed from the video to avoid brand effects. In the edited version, sentences were modified to communicate that adults should change their habits to improve their own health. Both original videos had not yet been exhibited in Chile.

### 2.3. Measures

In the interest of having a valid and reliable instrument, all scales used in this study were adopted from previous research [13,46,47,48,49,50,51,52,53]. Subjects were asked to fill in the questionnaire based on the scenario (fear or hope advertisement) presented a moment ago. Appendix A contains the measures included in the questionnaire. The dependent variables were healthy eating intention [46] and physical activity intention [47].

The manipulation check was an advertising message (fear vs. hope) [13]. Respondents rated the advertisement message on a bipolar anchored scale with “fear about changes in your weight for the future” and “hope for changes in your weight for the future” as endpoints. They responded to the following statement: “The advertisement message referenced what you would…” on a 7-point scale (−3 to +3). Higher numbers signal the hope advertisement message, while lower numbers indicate the fear advertisement message.

The independent variables were subjective norm (food) [48], subjective norm (exercise) [49], social influences regarding physical activity [47], self-efficacy [47], number of perceived barriers to a healthy lifestyle [47], past behavior (exercise) [50], past behavior (food) [46], frequency of fast food consumption [51], self-esteem [52], and self-control [53]. Finally, participants self-reported gender, age, marital status, height, weight, and perceived body weight (underweight, average weight, and overweight). The body mass index (BMI) was calculated with the reported height and weight data.

### 2.4. Hierarchical Linear Regression Analyses

The data were utilized in a series of hierarchical linear regression analyses to estimate the path coefficients for the hypothesized relationships. To minimize multicollinearity, the independent variables employed were mean-centered before creating the interaction terms. Regressions were estimated using the following equations to explain healthy eating intention and physical activity intention (the dependent variables, Y_i_) for each individual i:

(1) $$Y_{i}=\beta_{o}+\overset{15}{\underset{k=1}{\sum }}\beta_{k}\times X_{k,i}\;\left(\mathrm{Model}\;1\right)$$

(2) $$Y_{i}=\beta_{o}+\overset{15}{\underset{k=1}{\sum }}\beta_{k}\times X_{k,i}+\beta_{16}\times F_{i}\;\left(\mathrm{Model}\;2\right)$$

(3) $$Y_{i}=\beta_{o}+\overset{15}{\underset{k=1}{\sum }}\beta_{k}\times X_{k,i}+\beta_{16}\times F_{i}+\overset{15}{\underset{k=1}{\sum }}\beta_{16+k}\times X_{k,i}\times F_{i}\;\left(\mathrm{Model}\;3\right)$$

Model 1 includes in the regression the constant (β_o_) and k = 15 individual characteristics (frequency of fast food consumption, perceived body weight, past behavior (food), past behavior (exercise), subjective norm (food), subjective norm (exercise), social influences regarding physical activity, self-efficacy, self-esteem, self-control, number of perceived barriers to healthy lifestyle, female, age, married, and body mass index) for each individual i (X_k__,i_) as independent variables. Model 2 incorporates into the regression the binary variable that measures whether individual i was exposed to fear advertisement (F_i_ = 1) or hope advertisement (F_i_ = 0). Model 3 adds 15 interaction terms (F_i_ × X_k,i_).

## 3. Results

The specifics of the sample are detailed in Table 2. Women account for 48% of the sample and 29% of the sample is married. Table 3 and Table 4 exhibit the hypotheses test results. First of all, no multicollinearity existed among the employed constructs in this study, which can be confirmed through the variance inflation factors (VIFs) for each regression coefficient. They range from a low of 1.012 to a high of 3.731 in each regression, suggesting that they are at acceptable levels.

### 3.1. Manipulation Check for the Advertising Messages

An analysis of variance (ANOVA) was performed to ensure that the manipulation of the advertisement message was successful. As expected, there was a significant difference (F(1.281) = 23.641, *p* < 0.01) between the hope (M = 0.62) and the fear cases (M = −0.90) with means in the appropriate direction.

### 3.2. Regressions Predicting Healthy Eating Intention

As Table 3 summarizes, the Model 1 regression analysis results indicate that individual characteristics explain 43.7% of the variance in healthy eating intention. Adding the fear versus hope appeals in health advertisements in Model 2 increased the R^2^ value to 44.5% (ΔF = 3.997, *p* < 0.05). Consistent with Krishen and Bui [13], the results suggest that fear versus hope appeals in health advertisements have a more positive influence on healthy eating intention (*β* = 0.083, *p* < 0.05). That is, when subjects view a fear advertisement, they will be more likely to report greater healthy eating intention than when they view a hope-conditioned advertisement. Therefore, H1a is supported.

Adding the interaction terms in Model 3, using stepwise regression (15 interactions terms; fear advertisement × 15 individual characteristics) increased the R^2^ value to 47.2% (ΔF = 6.807, *p* < 0.01). The results suggest that the effect of fear advertisements on healthy eating intention is positively moderated by the frequency of fast food consumption (*β* = 0.083, *p* < 0.05). Therefore, H2a is supported.

The results also suggest that the effect of fear advertisements on healthy eating intention is negatively moderated by self-efficacy (*β* = −0.123, *p* < 0.01). Therefore, H7a is supported.

The results suggest that the effect of fear advertisements on healthy eating intention is not moderated by perceived body weight, past behavior, subjective norm, social influences, self-esteem, and self-control (ps > 0.05). Therefore, H3a, H4a, H5a, H6a, H8a, and H9a are not supported.

### 3.3. Regressions Predicting Physical Activity Intention

As summarized in Table 4, the Model 1 regression analysis results indicate that individual characteristics explain 29.2% of the variance in physical activity intention. Adding the fear versus hope appeals in health advertisements in Model 2 increased the R^2^ value by only 0.3% (ΔF = 1.175, *p* > 0.10). The results suggest that fear versus hope appeals in health advertisements have no main effect on physical activity intention (*p* > 0.10). Therefore, H1b is not supported.

Adding the interaction terms in Model 3, using stepwise regression (15 interactions terms; fear advertisement × 15 individual characteristics) increased the R^2^ value to 35.7% (ΔF = 6.381, *p* < 0.01). The results suggest that the effect of fear advertisements on physical activity intention is positively moderated by perceived body weight (*β* = 0.107, *p* < 0.05). Therefore, H3b is supported. The results suggest that the effect of fear advertisements on physical activity intention is positively moderated by past healthy eating behavior (*β* = 0.122, *p* < 0.01). Therefore, H4b is supported. The results also suggest that the effect of fear advertisements on physical activity intention is negatively moderated by subjective norm, food (*β* = −0.154, *p* < 0.01) and subjective norm, exercise (*β* = −0.187, *p* < 0.01). Therefore, H5b is supported.

The results suggest that the effect of fear advertisements on physical activity intention is not moderated by the frequency of fast food consumption, social influences, self-efficacy, self-esteem, and self-control (*p* > 0.05). Therefore, H2b, H6b, H7b, H8b, and H9b are not supported.

## 4. Discussion

Table 5 presents a summary of the results of the present study. The results in Chile are consistent with what was observed by Krishen and Bui [13] in the United States, showing that fear has a greater impact than hope in generating healthy eating intention. Consistent with previous research [17,18], the results also suggest that, when making health advertising, homogenous messages are not persuasive for heterogeneous audiences. Based on the new evidence found, it would be better to employ fear in cases where the audience is expected to have high past healthy eating behavior, high fast food consumption, or are perceived as overweight. Hope would be more effective in situations where the audience is expected to be high in subjective norms or self-efficacy.

The present study results suggest that fear and hope advertisements should be delivered considering the individual characteristics identified in the present study. Online channels (websites, social networks) easily allow audience segmentation using users’ interests and behaviors (e.g., fast food consumption, perceived body weight, past healthy eating behavior, self-efficacy). Therefore, policymakers and organizations concerned about people’s healthy eating and physical activity can use these online channels to send segmented messages (fear or hope appeals) to people using their own individual characteristics.

The frequency of buying fast food is an easy variable to measure and, for this reason, is useful for both companies and regulators to segment consumers [54,55]. The results suggest that fear messages should be directed towards frequent fast food consumers and hope messages towards less frequent fast food consumers in order to increase healthy eating intention. The results suggest that to increase healthy eating intention, fear can be communicated in warning messages accompanying the promotion or delivery of fast food [3,56]. In contrast, for people with a high level of self-efficacy, it is suggested to use hope appeals to increase healthy eating intention. This finding is consistent with Mowen et al. [45] who suggest that people with higher self-efficacy experience lower levels of fear.

Consistent with social support being a significant factor affecting a person’s eating and physical activity intentions and behaviors [35,36,37,38], the results also suggest that to increase people’s physical activity, hope messages should include the people who are important to the individual (subjective norm). In contrast, it is suggested to use fear appeals when the individual has a high level of perceived body weight (aversive state [28,29]) and past behavior (reinforcement [34]).

Several other topics are worth exploring in the future. Future research may analyze other advertisements (e.g., using fear and hope advertisements with the same theme, include a greater number of ads in varying themes), media (e.g., radio, print advertising, social media), emotions (e.g., love, guilt, pride, sadness, gratitude, shame), countries (e.g., European and Asian countries), and segments (e.g., children, adolescents). Such studies could increase the generalizability of the results as well as their applicability to health advertising. Considering that the observed effects may only be short-term, future research can also analyze the long-term effects (e.g., through panel data [57,58]) of the relationships proposed in this research. It would be interesting to analyze if, when repeating the stimuli over weeks, months, and years (e.g., repeating messages with fear appeals every week for a year), the effects on healthy eating intention and physical activity intention are maintained over time. Future research may also investigate the effects of mixing such stimuli (e.g., mixing, swapping ads with fear appeals with ads with hope appeals). Future research may also include other interesting and observable response variables such as the intention to recommend or share the health advertisement and behavioral variables (e.g., physical activity performed by the individual) or outcome (e.g., the weight of the individual).

## 5. Conclusions

Consistent with Krishen and Bui [13], the results suggest that in Chile, when comparing fear versus hope appeals in health advertisements, fear-conditioned advertisements have a greater positive impact on healthy eating intentions. In other words, when subjects view a fear advertisement, they will be more likely to report greater healthy eating intention than when they view a hope advertisement. This study also aimed to determine when, according to different individual characteristics, fear advertising works as a powerful tool for generating healthy eating and physical activity intention. The results suggest that the effect of fear advertisements on healthy eating intention is positively moderated by the frequency of fast food consumption and is negatively moderated by self-efficacy. The results suggest that fear versus hope appeals in health advertisements have no main effect on physical activity intention. However, the results suggest that the effect of fear advertisements on physical activity intention is positively moderated by perceived body weight and past healthy eating behavior and is negatively moderated by the subjective norm of food and subjective norm of exercise.

The results suggest that when making health advertising, standardized messages are not persuasive for diverse audiences. The present study results suggest that fear and hope appeals should be delivered considering the individual characteristics identified in the present study. Firms, governments, regulators, and other entities must consider these individual characteristics for an effective use of fear and hope appeals in the messages used to increase healthy eating intention and physical activity intention. Future research should also include these individual characteristics to analyze the effects of fear and hope appeals on healthy eating intention and physical activity intention.

## Figures and Tables

**Table 1:** Proposed hypotheses.

Variable	Hypothesis	Healthy Eating Intention (a)	Physical Activity Intention (b)
*Advertisement*			
Fear	H1	+	+
*Moderating role of individual characteristics*			
Fear × Frequency of fast food consumption	H2	+	+
Fear × Perceived body weight	H3	+	+
Fear × Past behavior	H4	+	+
Fear × Subjective norm	H5	−	−
Fear × Social influences regarding physical activity	H6	−	−
Fear × Self-efficacy	H7	−	−
Fear × Self-esteem	H8	−	−
Fear × Self-control	H9	−	−

Note: (+) indicates that the variable has a positive effect on the dependent variable and (−) indicates that the variable has a negative effect on the dependent variable.

**Table 2:** Descriptive statistics.

Variable	Mean	Standard Deviation	Minimum	Maximum
*Dependent variables*				
Healthy eating intention	4.00	0.88	1.00	5.00
Physical activity intention	3.25	0.79	1.00	4.00
*Individual characteristics*				
Frequency of fast food consumption	2.70	1.65	0.00	8.00
Perceived body weight	0.36	0.48	0.00	1.00
Past behavior—food	3.61	1.02	1.00	5.00
Past behavior—exercise	4.14	3.22	0.00	14.00
Subjective norm—food	5.22	1.69	1.00	7.00
Subjective norm—exercise	5.32	1.43	1.00	7.00
Social influences regarding physical activity	0.67	0.53	−1.00	1.00
Self-efficacy	7.67	2.19	0.00	10.00
Self-esteem	6.33	1.74	1.00	9.00
Self-control	4.71	1.46	1.00	7.00
Number of perceived barriers to healthy lifestyle	0.97	0.81	0.00	5.00
Female	0.48	0.50	0.00	1.00
Age	2.44	2.40	1.00	10.00
Married	0.29	0.45	0.00	1.00
Body mass index	23.83	3.33	15.78	33.84
*Advertisement and manipulation check*				
Fear	0.53	0.50	0.00	1.00
Advertising message (fear vs. hope)	−0.18	2.72	−3.00	3.00

**Table 3:** Hierarchical linear regression predicting healthy eating intention.

Variable	Hypothesis	Model 1	Model 2	Model 3
Intercept		4.000 **	4.000 **	4.002 **
*Individual characteristics (method = enter)*				
Frequency of fast food consumption		−0.043	−0.035	−0.031
Perceived body weight		0.028	0.014	0.058
Past behavior—food		0.349 **	0.353 **	0.337 **
Past behavior—exercise		0.100 *	0.102 *	0.121 *
Subjective norm—food		0.053	0.053	0.077
Subjective norm—exercise		0.089	0.084	0.068
Social influences regarding physical activity		0.004	0.008	0.010
Self-efficacy		0.192 **	0.191 **	0.177 **
Self-esteem		−0.092	−0.087	−0.089
Self-control		0.028	0.024	0.025
Number of perceived barriers to healthy lifestyle		−0.089	−0.101 *	−0.109 *
Female		0.166 **	0.173 **	0.169 **
Age		0.033	0.041	0.049
Married		0.084	0.078	0.068
Body mass index		−0.003	0.007	−0.042
*Advertisement (method = enter)*				
Fear	H1a: +		0.083 *	0.083 *
*Interaction effects (method = stepwise)*				
Fear × Frequency of fast food consumption	H2a: +			0.083 *
Fear × Self-efficacy	H7a: −			−0.123 **
Maximum VIF value		3.596	3.613	3.731
R^2^		0.437	0.445	0.472
Adjusted R^2^		0.405	0.411	0.436
R^2^ change		0.437	0.008	0.027
Partial F value		13.790 **	3.997 *	6.807 **
N		283	283	283

Note: Unstandardized regression coefficients are reported. * significant at *p* = 0.05, ** significant at *p* = 0.01.

**Table 4:** Hierarchical linear regression predicting physical activity intention.

Variable	Hypothesis	Model 1	Model 2	Model 3
Intercept		3.254 **	3.254 **	3.257 **
*Individual characteristics (method = enter)*				
Frequency of fast food consumption		−0.008	−0.004	0.011
Perceived body weight		0.047	0.040	0.034
Past behavior—food		0.016	0.018	0.048
Past behavior—exercise		0.068	0.069	0.050
Subjective norm—food		0.065	0.065	0.032
Subjective norm—exercise		0.061	0.058	0.057
Social influences regarding physical activity		0.079	0.081	0.082
Self-efficacy		0.310 **	0.309 **	0.297 **
Self-esteem		−0.021	−0.018	−0.006
Self-control		0.026	0.023	0.026
Number of perceived barriers to healthy lifestyle		−0.017	−0.024	−0.045
Female		0.044	0.048	0.061
Age		−0.143 **	−0.139 **	−0.128 *
Married		0.018	0.015	0.013
Body mass index		−0.029	−0.023	−0.006
*Advertisement (method = enter)*				
Fear	H1b: +		0.045	0.050
*Interaction effects (method = stepwise)*				
Fear × Perceived body weight	H3b: +			0.107 *
Fear × Past behavior—food	H4b: +			0.122 **
Fear × Subjective norm—food	H5b: −			−0.154 **
Fear × Subjective norm—exercise	H5b: −			−0.187 **
Maximum VIF value		3.596	3.613	3.662
R^2^		0.292	0.295	0.357
Adjusted R^2^		0.252	0.252	0.308
R^2^ change		0.292	0.003	0.063
Partial F value		7.331 **	1.175	6.381 **
N		283	283	283

Note: Unstandardized regression coefficients are reported. * significant at *p* = 0.05, ** Significant at *p* = 0.01.

**Table 5:** Summary of the study’s results

Variable	Hypothesis	Healthy Eating Intention (a)	Physical Activity Intention (b)
*Advertisement*			
Fear	H1	+	
*Moderating role of individual characteristics*			
Fear × Frequency of fast food consumption	H2	+	
Fear × Perceived body weight	H3		+
Fear × Past behavior	H4		+
Fear × Subjective norm	H5		−
Fear × Social influences regarding physical activity	H6		
Fear × Self-efficacy	H7	−	
Fear × Self-esteem	H8		
Fear × Self-control	H9		

Note: (+) indicates that the variable has a positive effect on the dependent variable and (−) indicates that the variable has a negative effect on the dependent variable. Blank spaces indicate that the hypotheses were not supported by the study’s results.

## Appendix A

Questionnaire (in order of appearance)

*Healthy eating intention (Chan et al. [46]; Cronbach’s alpha = 0.80)*

Do you intend to engage in healthy eating over the next week?

How likely is it that you will engage in healthy eating over the next week?

(5-point scale, 1 = definitely no, 5 = definitely yes)

*Physical activity intention (Hopman-Rock et al. [47])*

Do you plan to be more physically active in the short term? The answer categories were

1 = no, absolutely not, to 4 = yes, definitely.

*Advertising message (fear vs. hope) (adapted from Krishen and Bui [13])*

Respondents rated the advertisement message on a bipolar anchored scale with “fear about changes in your weight for the future” and “hope for changes in your weight for the future” as endpoints responding to the following statement: “The advertisement message referenced what you would…” on a 7-point scale (−3 to +3); higher numbers signal the hope-conditioned advertisement message while lower numbers indicate the fear advertisement message.

*Subjective norm-food (Conner et al. [48])*

People who are important to me think I should eat a healthy diet (unlikely–likely; scored 1 to 7)

*Subjective norm-exercise (Courneya [49])*

Most people who are important to me think I should engage in regular physical activity (7-point Likert scale, 1 = strongly disagree, 7 = strongly agree)

*Social influences regarding physical activity (Hopman-Rock et al. [47])*

How do you think people in your environment will react if you exercise more? Answer categories were: positive +1, neutral 0, negative-1.

*Self-efficacy (Hopman-Rock et al. [47])*

Do you think you will be able to be more physically active? The answer categories ranged from 0 = I am sure I cannot to 10 = I am sure I can.

*Number of perceived barriers to healthy lifestyle (adapted from Hopman-Rock et al. [47])*

A number of questions looked at several potential barriers: no time, no interest, not used to it, too expensive, no progress, feeling unhealthy, other problems. Answer categories were 1 = agree and 0 = disagree. Sum score for 7 questions.

*Past behavior-exercise (Abraham and Sheeran [50])*

How many days did you exercise in the last two weeks?

*Past behavior-food (Chan et al. [46])*

How often did you engage in healthy eating in the past month? (5-point scale, 1 = never, 5 = very often).

*Frequency of fast food consumption (Dunn et al. [51])*

Participants were asked to report the frequency of fast food consumption on a scale including responses: 0 = never, 1 = occasionally, 2 = once a month, 3 = once a fortnight, 4 = once a week, 5 = 2–3 times a week, 6 = 4–6 times a week, 7 = once a day, and 8 = more than once a day.

*Self-esteem (Robins et al. [52])*

I have high self-esteem (9-point Likert scale, 1 = strongly disagree, 9 = strongly agree)

*Self-control (Daly et al. [53])*

Self-control was measured using a single item where participants rated their level of self-control on a scale from 1 (little self-control) to 7 (disciplined).

*Female*

Male (0), Female (1)

*Age*

18–25 (1), 26–30 (2), 31–35 (3), 36–40 (4), 41–45 (5), 46–50 (6), 51–55 (7), 56–60 (8), 61–65 (9), +65 (10)

*Married*

Married (1), not married (0)

*Perceived body weight*

Underweight (0), average weight (0), overweight (1)

*Height*

Height in centimeters

*Weight*

Weight in kilograms

*Body mass index (BMI)*

The height and weight data were used to calculate the body mass index (BMI).
